# Genetic meta-analysis of twin birth weight shows high genetic correlation with singleton birth weight

**DOI:** 10.1093/hmg/ddab121

**Published:** 2021-05-06

**Authors:** Jeffrey J Beck, René Pool, Margot van de Weijer, Xu Chen, Eva Krapohl, Scott D Gordon, Marianne Nygaard, Birgit Debrabant, Teemu Palviainen, Matthijs D van der Zee, Bart Baselmans, Casey T Finnicum, Lu Yi, Sebastian Lundström, Toos van Beijsterveldt, Lene Christiansen, Kauko Heikkilä, Julie Kittelsrud, Anu Loukola, Miina Ollikainen, Kaare Christensen, Nicholas G Martin, Robert Plomin, Michel Nivard, Meike Bartels, Conor Dolan, Gonneke Willemsen, Eco de Geus, Catarina Almqvist, Patrik K E Magnusson, Hamdi Mbarek, Erik A Ehli, Dorret I Boomsma, Jouke-Jan Hottenga

**Affiliations:** 1 Avera Institute for Human Genetics, Avera McKennan Hospital and University Health Center, Sioux Falls, SD 57108, USA; 2 Department of Biological Psychology, Amsterdam Public Health Research Institute, Vrije Universiteit, Amsterdam, The Netherlands; 3 Department of Medical Epidemiology and Biostatistics, Karolinska Institutet, Stockholm, Sweden; 4 MRC Social, Genetic and Developmental Psychiatry Centre, Institute of Psychiatry, Psychology and Neuroscience, King's College London, London, UK; 5 Genetic Epidemiology Laboratory, QIMR Berghofer, Brisbane, Queensland, Australia; 6 The Danish Twin Registry, Department of Public Health, University of Southern Denmark, Odense, Denmark; 7 University of Helsinki, Institute for Molecular Medicine Finland (FIMM), Helsinki, Finland; 8 Institute for Molecular Bioscience, The University of Queensland, Brisbane, Queensland, Australia; 9 Gillberg Neuropsychiatry Centre, Institute of Neuroscience and Physiology, University of Gothenburg, Gothenburg, Sweden; 10 Department of Clinical Immunology, Copenhagen University Hospital, Rigshospitalet, Copenhagen, Denmark

## Abstract

Birth weight (BW) is an important predictor of newborn survival and health and has associations with many adult health outcomes, including cardiometabolic disorders, autoimmune diseases and mental health. On average, twins have a lower BW than singletons as a result of a different pattern of fetal growth and shorter gestational duration. Therefore, investigations into the genetics of BW often exclude data from twins, leading to a reduction in sample size and remaining ambiguities concerning the genetic contribution to BW in twins. In this study, we carried out a genome-wide association meta-analysis of BW in 42 212 twin individuals and found a positive correlation of beta values (Pearson’s *r* = 0.66, 95% confidence interval [CI]: 0.47–0.77) with 150 previously reported genome-wide significant variants for singleton BW. We identified strong positive genetic correlations between BW in twins and numerous anthropometric traits, most notably with BW in singletons (genetic correlation [*r*_g_] = 0.92, 95% CI: 0.66–1.18). Genetic correlations of BW in twins with a series of health-related traits closely resembled those previously observed for BW in singletons. Polygenic scores constructed from a genome-wide association study on BW in the UK Biobank demonstrated strong predictive power in a target sample of Dutch twins and singletons. Together, our results indicate that a similar genetic architecture underlies BW in twins and singletons and that future genome-wide studies might benefit from including data from large twin registers.

## Introduction

Birth weight (BW) is a powerful predictor of infant and newborn survival, with lower weight infants being at higher risk of mortality (1–3). BW is also associated with a wide array of health-related variables in later life (4), with varying effect sizes, including adult body mass index (BMI) (5,6), cardiovascular disease (7,8), type 2 diabetes (9), hypertension (10–12) and psychological distress (13). Our knowledge of the biological pathways underlying BW is growing with the rapidly increasing number of genetic variants identified in genome-wide association (GWA) studies. Yet, these investigations mainly focus on BW in singletons and tend to exclude data from twins in the discovery analysis. Therefore, knowledge about the genetic overlap between BW in singletons and twins is limited and it is not clear to what degree findings in singletons can be generalized to twins and to what extent data from twins can contribute to gene discovery for BW. This knowledge would be useful as a considerable genetic overlap would indicate that data from singletons and twins could be combined for attaining larger sample sizes.

BW is a complex and multifactorial trait (14,15). Maternal and fetal genomes conjointly determine fetal size, making estimations of the heritability of BW challenging as offspring and maternal genomes are not independent. In twins, BW is different from BW in singleton births because of their lower gestational age. The main factor explaining lower gestational age is uterine overdistension (16). Still, twin and family studies suggest similar heritability estimates for BW, ranging from 10 to 40% (17–20), indicating a moderate contribution of genetic factors to BW variation. Of interest for our quest is a study from the Netherlands in which heritability was estimated from data on parents and their singleton offspring and from data on mono- and dizygotic twins (19). The heritability estimates for BW and height were all around 0.3 and highly comparable in both groups.

The number of genetic variants identified for BW is growing based on findings from GWA studies (GWAS). In a 2010 study by Freathy et al. (21), two variants, in *ADCY5* and near *CCNL1*, were found to influence variation in BW in singletons. The number of associated variants increased to seven in 2013 with an expanded meta-analysis study of over 69 000 European individuals (22). In a multi-ancestry GWA meta-analysis (GWAMA) by Horikoshi and colleagues (23), BW and genotype data were collected for 153 781 singletons. The result of this effort was the identification of 59 independent signals, capturing approximately 15% of the variance in BW. Beaumont and colleagues (24) also examined the contribution of fetal versus maternal genetic effects and identified ten maternal loci influencing offspring birthweight. Additional GWA efforts have been undertaken to ascertain the maternal and fetal genetic effects on BW and their relation to cardiometabolic risk, in which 190 independent associations were discovered (25). To date, only one GWA study has been performed on BW in twins (4593 female twins from the UK), which identified one variant on chromosome 9, close to the *NTRK2* gene (26).

The Developmental Origins of Health and Disease (DOHaD) hypothesis is based on observations that adverse influences early in development, particularly in the intrauterine environment, result in permanent physiological and metabolomic changes leading to increased risk of disease in adulthood (27–29). One hypothesis, postulated by Barker in the 1990s, proposed that intrauterine growth restriction, low BW and premature birth have a causal relationship to hypertension, coronary heart disease and non-insulin-dependent diabetes in later life. Barker and colleagues traced infant mortality rates in England during the early 1900s and found strong geographical relations between infant death and high rates of mortality resulting from coronary heart disease years later (27). They postulated that the geographic associations of infant mortality and adult death rates ‘reflects variations in nutrition in early life, which are expressed pathologically on exposure to later dietary influences’ (p.1081). At the time, the typical certified cause of death in newborn babies was low BW. Thus, the hypothesis was that low BW babies surviving infancy suffered from fetal undernutrition, exhibiting non-communicable changes in metabolism and physiology, in turn, increasing coronary heart disease risk in adulthood (30). Low BW can serve as a proxy for a suboptimal intrauterine environment and is not only associated with cardiovascular disease (31), but also with respiratory disease (32), various psychiatric disorders (33), as well as mental health, cognitive and socioeconomic outcomes (34).

In general, the DOHaD and the Barker hypotheses are environmentally based. That is, the existence of an adverse intrauterine environment leads to decreased BW and long-term cardiometabolic sequalae in offspring. Alternatively, strong genetic correlations between low singleton BW and indicators of metabolic and cardiovascular health, as described in the meta-analysis by Horikoshi and colleagues (23), correspond more closely to the Fetal Insulin Hypothesis (35). In this context, the correlations between BW and cardiometabolic disorders are driven by the transmission of maternal genes to the offspring. However, genetic correlations between BW and the cardiometabolic traits could be driven through the fetal and/or the maternal genome. The latter is broadly consistent with the DOHaD/Barker hypothesis since the maternal genome defines the intrauterine environment, whereas the former more likely reflects the Fetal Insulin Hypothesis (36). Recent studies have investigated these differences in hopes of disentangling the relative contributions of fetal and maternal effects on BW and later life cardiometabolic disease (25,37).

On average, twins have lower BW than singletons since twin pregnancy is characterized by a shorter gestational duration (16) and because fetal growth slows down after approximately 32 weeks of gestation (38–41). Therefore, investigations into the genetic architecture of BW and other birth-related characteristics often exclude twins, even though this may lead to a significant decrease in sample size. Concerning the DOHaD hypothesis, there is no evidence that the relation between BW and later-life disease differs between twins and singletons as demonstrated for blood pressure or anti-hypertensive drug use (42–44) and diabetes (45,46).

This study aimed to search for common genetic variants underlying BW in twins by carrying out a meta-analysis of genetic association studies in twins and compare the results to those for BW in singletons. To this end, four approaches were employed: 1) A meta-analysis of combined GWA results from five European twin cohorts, UK Biobank, one Australian twin cohort and one twin cohort from the Midwestern region of the United States of America. 2) An assessment of the genetic correlations between BW in twins and BW in singletons. 3) The evaluation of the genetic correlations between BW in twins and a range of traits and diseases in later life, including anthropometric and neuropsychiatric characteristics. 4) An assessment of the predictive performance of BW polygenic scores in twins and singletons.

## Results

### Meta-analysis

**Figure 1:**
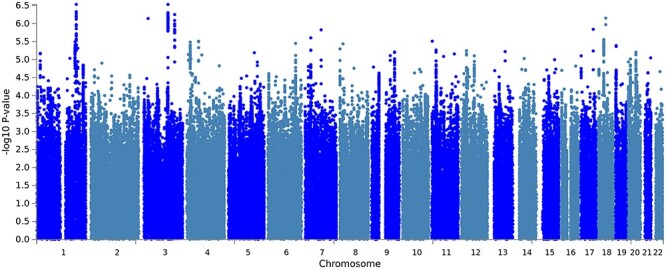
Manhattan plot from the genome-wide association meta-analysis for BW. The association *P*-value (on -log_10_ scale) for each of up to 7 692 335 SNPs (*y*-axis) is plotted against the genomic position according to NCBI Build 37 (*x*-axis). For plotting purposes, overlapping data points are not drawn for filtered SNPs with a *P*-value ≥ 1 × 10^–5.^

We carried out a GWAMA for BW in 42 212 twins. The meta-analysis QQ-plot, showing the expected distribution of genome-wide *P*-values compared to the observed values across SNPs, can be found in Supplementary Material, Figure S1. The Manhattan plot for the meta-analysis is shown in Figure 1. There were no genome-wide significant SNPs at the defined minimum *P*-value for lead SNPs (*P* < 5 × 10^−8^); however, two lead SNPs had an association signal of *P* < 5 × 10^−7^. These SNPs were located on chromosome 1 (rs10800682, hg19 position 1:200198946, *P* = 2.92 × 10^−7^) and chromosome 3 (rs3845913, hg19 position 3:123100606, *P* = 2.93 × 10^−7^). rs10800682 is independent (>12 Mb, EUR r^2^<0.05) of all genome-wide significant loci found by Horikoshi and colleagues (23). rs3845913 is an intronic variant of ADCY5 and is ∼31 kb downstream of rs11719201 (EUR r^2^ 0.154), one of 60 loci previously associated with BW (23).

### Replication of previous association results

**Figure 2:**
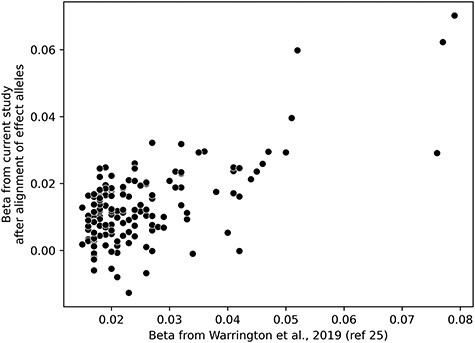
Scatter plot of the beta estimates from the overlapping SNPs between the current study and those reported in Warrington et al. (25) for the GWAS on own BW (*P* < 6.6 × 10^−9^). Of the significant SNPs, 150 overlapped with the current study.

Though no genome-wide significant SNPs were identified, we evaluated the performance of SNPs in the current study with the genome-wide significant SNPs signals (*P* < 6.6 × 10^−9^) recently identified by Warrington et al. (25) in a GWAS of own BW. Of the significant SNPs, 150 overlapped with the current study after retention of markers present in greater than 70% of all study participants. As shown in Figure 2, following alignment of effect alleles, the beta estimates between overlapping markers are highly correlated (Pearson’s *r* = 0.66, 95% CI: 0.47–0.77). Summary statistics of the 150 overlapping variants are presented in Supplementary Material, Table S1. Overall, the positive linear relationship indicates that the previously reported significant variants behave similarly between singletons and twins.

Additionally, since gestational age was not available in all cohorts, we assessed heterogeneity of the overlapping SNPs mentioned above (i.e. 150) using METAL (implemented as Cochran’s *Q*-test). No significant heterogeneity in allelic effects was observed after Bonferroni correction (*P* > 0.00033). The smallest reported *P*-value of heterogeneity statistics in the current study was 0.002, which is in line with the smallest reported *P*-value of heterogeneity statistics for the genome-wide significant variants reported in Warrington et al. of 0.004 (Supplementary Material, Table S1).

### Genetic correlations

**Figure 3:**
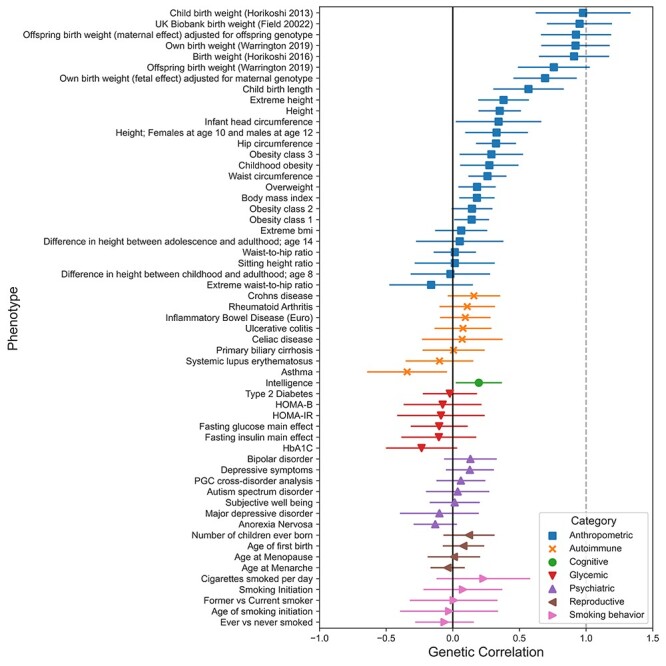
Genetic relationships between BW in twins and 57 other phenotypes. SNP-based genetic correlations (*r*_g_) between BW in twins and a range of other traits and diseases using LD Score regression. The bars represent 95% confidence intervals. The genetic correlation estimates are color coded according to their respective category. HbA1C = hemoglobinA1C, HOMA-IR = homeostatic model assessment of insulin resistance, HOMA-B = homeostatic model assessment of beta cell function, PGC=Psychiatric Genetics Consortium, BMI = body mass index. PubMed reference numbers (PMID) for each trait are listed in Supplementary Table 2.

The results from the genetic correlation analyses of BW in twins can be found in Figure 3 and Supplementary Material, Table S2. In general, the strongest genetic correlations were with anthropometric traits, specifically BW-related phenotypes. Previous studies have investigated and attempted to partition maternal and fetal genetic effects on BW, allowing comparisons to individual and parental effects in this study.

The strongest genetic correlation was with 'child birth weight' (i.e. the individuals own genetic effect on their BW) (genetic correlation [*r*_g_] = 0.98, 95% confidence interval [CI]: 0.62–1.33) based on a discovery GWAS of 26 836 European individuals (22). Similarly, robust positive correlations were found with other phenotypes of the individuals own genetic effect on their BW, including UK Biobank birth weight (data field 20 022) (*r*_g_ = 0.95, 95% CI: 0.71–1.19), 'own birth weight' (*r*_g_ = 0.92, 95% CI: 0.66–1.18) derived from an expanded GWAS of 286 870 European individuals (25) and 'birth weight' (*r*_g_ = 0.91, 95% CI: 0.65–1.17) in 143 677 European individuals (23). It is important to note that genetic correlations referenced above are from three studies that are not entirely independent. Sequential studies (in chronological order, references (22,23,25)) used a core set of samples obtained by the Early Growth Genetics Consortium (EGG), which were expanded upon with new releases of the UK Biobank.

A positive correlation was also observed with 'offspring birth weight' (i.e. the maternal genetic effect on offspring BW), as measured in 216 611 mothers (25) (*r*_g_ = 0.76, 95% CI: 0.49–1.03). Of the genetic correlations with other phenotypes, six additional anthropometric traits exhibited strong positive genetic correlations, including offspring birth weight (maternal genetic effect on offspring BW after adjusting for the correlated offspring’s genotype) (*r*_g_ = 0.92, 95% CI: 0.66–1.19), own birth weight (individuals own genetic effect on their own BW after adjusting for the correlated maternal genotype) (*r*_g_ = 0.69, 95% CI: 0.45–0.93), child birth length (*r*_g_ = 0.57, 95% CI: 0.30–0.83), extreme height (*r*_g_ = 0.38, 95% CI: 0.19–0.57), height (*r*_g_ = 0.35, 95% CI: 0.19–0.51) and hip circumference (*r*_g_ = 0.32, 95% CI: 0.17–0.47).

Glycemic traits were all negatively associated with BW, whereas cognitive characteristics, measured by intelligence, correlated positively (*r*_g_ = 0.20, 95% CI: 0.02–0.37). Genetic correlations of BW in twins with autoimmune disorders, psychiatric disorders, reproductive traits and smoking behavior yielded mixed results.

The SNP heritability (*h*^2^) was calculated using LD Score regression. The *h*^2^ was estimated to be 0.0407 for BW in twins. For BW in singletons, the heritability estimates from three studies were *h*^2^ = 0.1139, *h*^2^ = 0.0985 and *h*^2^ = 0.1016 for ‘child birth weight’ (22), ‘own birth weight’ (25) and ‘birth weight’ (23), respectively. The heritability estimate of UK Biobank birth weight was *h*^2^ = 0.1006.

### PolyGenic Score prediction

The PGS, based on summary statistics from GWA analyses of BW in UK Biobank, robustly predicted BW in NTR twins and singletons. The PGS including the fraction of SNPs with a *P*-value selection threshold of 0.01 was the best predictor for BW in twins (β = 68.19, *P* = 2.10 × 10^−51^, PGS *R*^2^ = 0.02) and singletons (β = 108.18, *P* = 6.94 × 10^−57^, PGS *R*^2^ = 0.03), as shown in Table 1.

**Table 1:** Results of the PGS prediction in NTR twins and singletons

	Twins (*N* = 10 487)				Singletons (*N* = 6892)			
Prop	β_PGS_	SE_PGS_	P_PGS_	PGS *R*^2^	β_PGS_	SE_PGS_	P_PGS_	PGS *R*^2^
0.001	18.89	4.66	5.04E-05	0.00	34.28	6.87	6.09E-07	0.00
0.003	19.94	4.57	1.26E-05	0.00	38.67	6.72	8.77E-09	0.00
0.005	54.19	4.72	1.86E-30	0.01	75.01	6.75	1.13E-28	0.02
0.01	68.19	4.52	2.10E-51	0.02	108.18	6.81	6.94E-57	0.03
0.05	60.35	4.50	5.39E-41	0.01	101.71	6.88	1.73E-49	0.03
0.1	58.48	4.50	1.26E-38	0.01	99.71	6.88	1.52E-47	0.03
0.2	57.25	4.50	4.46E-37	0.01	98.45	6.89	2.24E-46	0.03
0.3	56.83	4.50	1.43E-36	0.01	98.10	6.89	4.96E-46	0.03
0.5	56.53	4.50	3.23E-36	0.01	97.77	6.89	1.01E-45	0.03
INF	55.39	4.53	2.09E-34	0.01	90.48	6.98	1.92E-38	0.02

Note: Prop (proportion) is the *P*-value fraction for SNP inclusion in the polygenic score (PGS), β is the regression coefficient for each term with standard error (SE) and *P*-value (P). PGS *R*^2^ is the phenotypic variance explained by the PGS.

**Figure 4:**
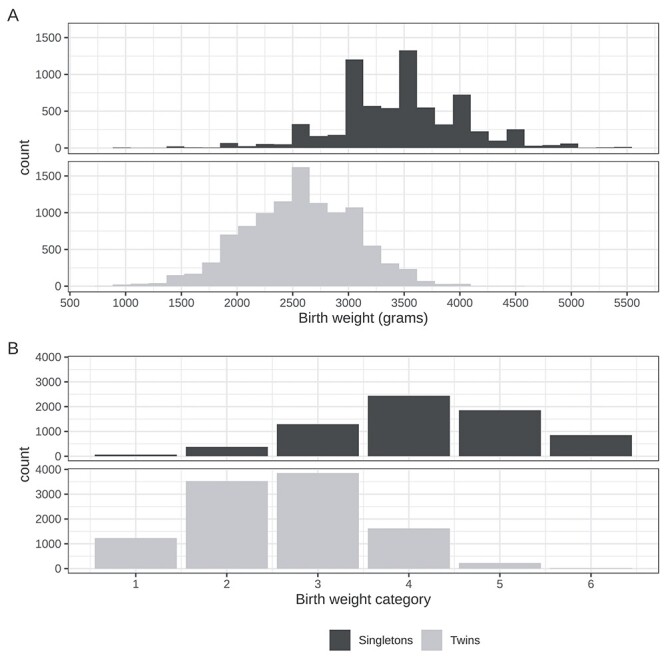
Histograms of raw and categorical BW for NTR twins and singletons. Panel A shows histograms for raw BW in grams. Panel B portrays the distributions for BW categories 1–6 as described in the text. *N* = 10 487 twins; 6892 singletons. It is of note to point out the peaks corresponding to ~500 g increments in the singletons in panel A, which simply may reflect assessment of BW measures in this group.

As shown in Figure 4A, a comb-like distribution of raw BW was observed in singletons, corresponding to even ~500 g increments, reflecting assessment of BW in this group.

BW category was also evaluated as the response variable (histograms in Figure 4B). The evaluation was done in all target samples (twins and singletons) by including twin status and an interaction of PGS and twin status as predictors in the model (Table 2). As before, the PGS including the fraction of SNPs with a *P*-value selection threshold of 0.01 represented the best predictor of BW category (β = 0.18, *P* = 1.68 × 10^−49^, PGS R^2^ = 0.02). Together, the results of PGS prediction analyses suggest that BW PGS constructed from a large representative discovery population predict BW similarly in a target population of twins and singletons.

**Table 2:** Results of the PGS prediction of BW category for NTR twins and singletons (*N* = 17 379)

Prop	β_PGS_	SE_PGS_	P_PGS_	β_TS_	SE_TS_	P_TS_	β_INT_	SE_INT_	P_INT_	PGS *R*^2^
0.001	0.06	0.01	2.06E-06	–1.07	0.02	<0.001	–0.03	0.01	0.09	0.00
0.003	0.06	0.01	2.69E-07	–1.07	0.02	<0.001	–0.02	0.01	0.09	0.00
0.005	0.12	0.01	3.74E-23	–1.07	0.02	<0.001	–0.03	0.01	0.06	0.01
0.01	0.18	0.01	1.68E-49	–1.07	0.02	<0.001	–0.06	0.01	6.24E-05	0.02
0.05	0.17	0.01	1.48E-42	–1.07	0.02	<0.001	–0.06	0.01	3.23E-05	0.01
0.1	0.17	0.01	6.58E-41	–1.07	0.02	<0.001	–0.06	0.01	3.09E-05	0.01
0.2	0.17	0.01	7.29E-40	–1.07	0.02	<0.001	–0.06	0.01	2.98E-05	0.01
0.3	0.17	0.01	1.44E-39	–1.07	0.02	<0.001	–0.06	0.01	2.83E-05	0.01
0.5	0.17	0.01	2.68E-39	–1.07	0.02	<0.001	–0.06	0.01	2.83E-05	0.01
INF	0.15	0.01	3.21E-33	–1.07	0.02	<0.001	–0.05	0.01	<0.001	0.01

Note: Prop (proportion) is the *P*-value fraction for SNP inclusion in the polygenic score (PGS), β is the regression coefficient for each term with SE and *P*-value (*P*) for the PGS, TS and the INT of TS and PGS. PGS *R*^2^ is the phenotypic variance explained by the PGS.

## Discussion

We performed a genome-wide meta-analysis of BW in twins and compared the genetic architecture of BW between twins and singletons. Our results, particularly the genetic correlation and PGS analyses, provide compelling evidence for considerable genetic overlap between BW in twins and singletons.

The genetic correlation between BW in twins and the most recent reported results in singletons was very strong (*r*_g_ = 0.92, 95% CI: 0.66–1.18), indicating a large overlap in the genetic variants influencing BW in the two groups. The genetic associations with health-related traits, when comparing the size and direction from our genetic correlation analyses with the results from Horikoshi and colleagues (23), showed remarkably similar results. This similarity suggests that the differential pattern of fetal growth between twins and singletons does not affect the relation between BW and later-life disease.

We evaluated the predictive performance of PGS derived from a GWAS on BW from a large representative population from the UK Biobank in a large target sample of NTR twins and non-twins. The PGS calculated from the proportion of SNPs with a *P*-value selection threshold of 0.01 demonstrated robust prediction in both singletons (*P* = 6.94 × 10^−57^) and twins (*P* = 2.10 × 10^−51^). While the proportion of variation explained by the best predicting PGS was small for twins at 2% and non-twins at 3%, despite moderate heritability estimates, such PGS represents common genetic architecture underlying BW in twins and singletons even though there are clear differences in BW between the two groups. Smaller heritability estimates were also observed for BW in twins, potentially indicating a form of sibling competition. That is, if one twin grows and occupies the growing space of the co-twin, the genes that increase the BW of the larger twin may also limit the growth of the co-twin. Consistent with our results, sibling competition would result in a dampened effect of the PGS and would be reflected in lower heritability estimates in twins.

The results of the GWAMA did not yield SNPs significantly associated with BW in twins. Two lead SNPs, rs10800682 and rs3845913, had association signals of *P* < 5 × 10^−7^. rs10800682 was not near (>2 Mb away) and was independent (*r*^2^ < 0.05) of all genome-wide significant loci found by Horikoshi and colleagues (23), making it a potential candidate for future twin studies. rs3845913 is an intronic variant of *ADCY5*, which, along with *CCNL1*, were two of the first genes robustly associated with fetal growth and BW (21). Additionally, rs3845913 is ~31 kb downstream and is in LD (*r*^2^ = 0.154) with rs11719201 (an intronic variant of *ADCY5*), one of 60 loci previously associated with BW (23). To pinpoint exactly how and through which gene(s) rs10800682 and rs3845913 may affect BW, additional and functional follow-up studies are necessary. Previously associated alleles at *ADCY5* were found to be BW lowering and risk increasing for type 2 diabetes, consistent with the fetal insulin hypothesis (35).

The results from this study strongly suggest that BW data from twins and singletons may be meta-analyzed together in GWAMA, despite the limited sample size of the discovery GWAMA in twins (*N* = 42 212). Another limitation is that we corrected for birth order, gestational age and maternal age at birth in a majority of cohorts but could not do so for all cohorts due to data availability. This information should ideally always be included when BW data are collected.

Additionally, we report genome-wide estimates of shared genetic effects based on common genetic variation (SNPs with MAF > 0.01 per default settings in LDHub). Suppose the effects of rare variants are not shared similarly to the effects of common variants for each phenotype comparison. In that case, the genetic correlation estimates could be misleading. However, in terms of their shared influences on pairs of phenotypes, there is not a theoretical reason to expect systematic differences in the effects of rare and common variants. Rare variants with larger effects would not preclude carrying far more numerous common variants with smaller effects. Thus, the genetic correlations presented in this study may provide reasonable estimates based on common genetic variation; however, to validate these findings, rare variant studies are needed. Future studies may also expand upon our genetic correlation estimates by utilizing non-European populations, greater sample sizes (for discovery and trait-specific phenotypes as they become available) and increased density across the genome.

Concerning the results of the PGS prediction, we note that the *P*-value selection threshold of the most predictive PGS is a function of the effect size distribution, the statistical power of the discovery GWAMA and the NTR target data, the genetic architecture of BW, as well as the fraction of associated markers. 

Follow-up research may aim to better understand BW since it is influenced by direct fetal and indirect maternal genetic influences through the intra-uterine environment. The amount of variance in BW explained by the maternal genotype has been estimated as substantially smaller than the fetal genetic contribution (47). Recent work suggests that fetal size measurements at birth are predominantly determined by the fetal genome, whereas the gestational duration is primarily dictated by the maternal genome (48). A better understanding of the genetic architecture of BW, and fetal growth more generally, will aid in the elucidation of immediate health outcomes (e.g. preterm birth, fetal growth restriction) and reveal relationships with later-life health outcomes (e.g. cardiovascular disease, type-2 diabetes).

To conclude, we show that based on genetic correlation and PGS analyses, the genetic architecture of BW in twins and singletons is similar. Of course, it is known that mean differences in BW between twins and singletons exist; however, the findings of this work strongly suggest that the genetic causes of variation are the same. Bearing this in mind, the results of this work indicate that it is appropriate to meta-analyze twins and singletons for genetic studies of BW. However, careful consideration of analytical strategies will be needed since details specific to twins may not apply to full-term singletons. Small groups of twins might still need to be excluded; for example, the highly discordant BW pairs due to the possibility for twin-to-twin transfusion syndrome (TTTS). Also, in full-term singletons, a typical gestational age cut-off for exclusion (e.g. born before 37 weeks) is often applied, which will not be applicable with the inclusion of twins due to shorter gestational duration (16) and delayed fetal growth after 32 weeks (38–41). One approach to address these issues would be to perform separate GWAS on standardized BW in each group with appropriate exclusion criteria and covariates specific to twins and non-twins with subsequent meta-analysis of *P*-values since beta estimates and intercepts will be affected by raw differences in BW.

## Materials and Methods

### Samples

Eight population-based twin registers supplied data: the Netherlands Twin Register (NTR) (49,50), Queensland Institute of Medical Research (QIMR—comprised of the Queensland Twin Registry (51) and the Australian Twin Registry (52,53)), Danish Twin Registry (DTR) (54), Finnish Twin Cohort Study (FinnTwin) (55,56), Twins Early Development Study (TEDS) (57), Child and Adolescent Twin Study in Sweden (CATSS) (58–60), Avera Twin Register (ATR) (61,62) and the UK Biobank (UKB) (63). In UKB, twins were identified as previously described (64). A detailed description of cohort sample characteristics can be found in Table 3. Information on genotyping and quality control procedures for each cohort can be found in Supplementary Material, Table S3.

**Table 3:** Number of individuals, birth weight and associated measures per cohort

Cohort	Country	Sample size (M/F)	Mean (SD) BW (grams)	Birth year range	Mean (SD) maternal age (years)	Mean (SD) gestational age (weeks)	Data collection
AVERA	USA	279 (88/191)	2431.97(547.42)	1939–2018	29.09 (4.92)	36.75 (2.92)	Self-report, parent-report
CATSS	Sweden	13 595 (6706/6889)	2651.83 (564.34)	1985–2005	30.72 (4.62)	36.54 (2.64)	Medical birth registry
DTR	Denmark	1432 (687/745)	2688.80 (534.10)	1903–1952	NA	NA	Mid-Wife records and self-report
FinnTwin	Finland	1778 (812/966)	2749 (448.73)	1974–1987	29.21 (4.63)	37.36 (1.81)	Parent-report
NTR	The Netherlands	6951 (2942/4009)	2586.16 (467.62)	1922–2012	30.00 (4.33)	37.14 (2.04)	National youth health services, self-report and parent-report
QIMR	Australia	5435 (2263/3172)	2626.53 (510.54)	1922–1999	29.34 (5.04)	37.90 (2.14)	For birthweight and gestational age: Self-report or parental report depending on study (for adults); maternal report (for adolescents). For gestational age: assumed 37 weeks if not available. For birth year and maternal age: derived from dates of birth.
TEDS	UK	6527 (3109/3418)	2522.25 (530.86)	1994–1996	31.01 (4.79)	36.47 (2.41)	Parent-report
UKB	UK	6215 (2300/3915)	2431.64 (737.42)	1937–1970	NA	NA	Self-report (UKB ID 20022)

CATSS=  Child and Adolescent Twin Study in Sweden, DTR = Danish Twin Registry, NTR = Netherlands Twin Register, QIMR  =  Queensland Institute of Medical Research, TEDS  =  Twins Early Development Study, and UKB = UK Biobank. M/F are counts of male and female individuals, respectively. SD is standard deviation. NA represents unavailable information.

### Study-level analyses

Birth weight (BW) measures were *z*-score transformed ([BW_value_−BW_mean_]/BW_standard deviation_) before analysis. Each participating study group performed the association analyses between each SNP genotype and BW *z*-scores with the following covariates where available: sex, gestational age, year of birth, maternal age at birth, birth order and relevant study-specific metrics (e.g. principal components (PCs) correcting for genomic ancestry). For all cohorts, except ATR, birth order was available. The analysis was performed without adjustment for maternal age at birth and gestational age in the DTR. Association analyses were performed in PLINK *v1.07* (65) with the Generalized Estimation Equation (GEE) package using the R-package plugin to correct for family relatedness or according to local best practices (details provided in Supplementary Material, Table S3). Sample exclusion criteria were phenotypic outliers (BW z-score greater than or less than five standard deviations from the mean), premature births (gestational age less than 33 weeks), monozygotic (MZ) twins with TTTS including twin pairs with BW more than 35% discordant (a group likely including TTTS twins), triplets and higher-order multiple births and participants with non-European ancestry.

### Meta-analysis

Summary statistics from each cohort GWA analysis underwent another round of standard quality control before meta-analysis. The R-package EasyQC (66) was used to perform quality control analyses. Insertions and deletions, SNPs with missing or invalid values, markers with Minor Allele Frequency (MAF) < 0.01, and those with poor imputation quality (<0.30) were excluded. Resulting quality controlled summary statistics from each cohort were meta-analyzed using the inverse variance-based approach in METAL (67). Genomic control was applied to adjust the statistics generated by each cohort (68). In the meta-analysis, SNPs present in greater than 70% of all participants were retained.

### Association tests

FUMA (FUnctional Annotation and Mapping *v1.3.6*) (69) was used to annotate GWAMA results and identify genomic risk loci. These loci were defined as independent lead SNPs exhibiting maximum distance between their linkage-disequilibrium (LD) block. For genome-wide significance in the meta-analysis, a *P*-value threshold of 5 × 10^−8^ was adopted. The minimum threshold for defining independent significant SNPs was *r*^2^ ≥ 0.6, which was used to determine the borders of the genomic risk loci. The minimum threshold for defining lead SNPs, used for clumping of the independent significant SNPs, was *r*^2^ ≥ 0.1. Independent significant SNPs closer than 250 kb were merged into one genomic risk locus. SNPs in LD with the independent significant SNPs were considered candidate SNPs and defined the borders of the genomic risk loci. We tested whether the signals from our analyses overlap with previously identified loci for BW in singletons. In agreement with Horikoshi et al. (23), if a lead SNP mapped >2 Mb away from, and was statistically independent (LD *r*^2^ < 0.05 based on European population reference set) of any of the 60 previously identified loci, it was considered novel. We calculated the *r*^2^ between the signals with the web-based application LDmatrix within the LDlink (*v3.8*) (70) suite of tools.

### Genetic correlations

To quantify the degree of shared genetic contribution between BW in twins and BW in singletons and to correlate BW in twins to other individual-level health-related traits and diseases, we employed LD Hub (*v1.9.3*) (http://ldsc.broadinstitute.org/ldhub/) (71). LD Hub is a centralized database of summary-level GWA study results facilitating the calculation of genetic correlations (72) between user-supplied summary statistics and a variety of user-selected traits using LD score regression (73). HapMap3 SNPs from summary statistics of the GWAS for each trait and pre-computed LD scores were used in the analyses (available on: https://github.com/bulik/ldsc). LD score regression requires large sample sizes and utilizes LD information from an ancestry-matched reference panel; therefore, genetic correlation analyses were constrained to European GWA study samples. SNPs with a MAF $\le$ 0.01 were excluded.

For the comparisons with previous genome-wide genetic correlation analyses in singletons (7), we selected the following categories of traits: anthropometric traits, reproductive traits, glycemic traits, autoimmune disorders, cognitive abilities, psychiatric diseases and smoking behavior. In total, we tested for association with 57 traits.

SNP heritability (*h*^2^) was calculated in LD Hub with LD score regression to evaluate how much of the variation in BW could be ascribed to common additive genetic variation.

### PolyGenic Score prediction

GWAS results on BW from the UK Biobank (data field 20022) (http://www.nealelab.is/uk-biobank/) served as the discovery set for calculating polygenic scores (PGS) in the NTR target dataset. For the PGS prediction of BW in the NTR, participants with complete BW data and maximum information on covariates (genomic PCs, sex, year of birth, gestational age, twin status and genotyping platform) were included. When not available, gestational age was imputed with the mean gestational age separately for twins (mean = 37.38 weeks) and singletons (mean = 39.89 weeks). Genotyping platform and ten genomic PCs were included in the model to account for batch effects (i.e. non-random selection of samples genotyped on specific arrays) and residual population stratification. The target sample consisted of 17 379 individuals, comprising 10 487 twins and 6892 singletons. Summary statistics from the UK Biobank GWAS on BW were adjusted for the effects of LD with LDpred (74) using the LD structure of European populations in the 1000 Genomes references set (75). Recalculated effect size estimates representing ten fractions of *P*-value significance (0.001, 0.003, 0.005, 0.01, 0.05, 0.1, 0.2, 0.3, 0.5, INF (infinitesimal)) were used for allelic scoring in PLINK (65).

We used the PGS to predict BW in NTR twins and singletons using GEE methods in R (76), taking into account familial relationships. We also evaluated the predictive performance of the PGS on categorical BW in the entire target sample of twins and singletons by including twin status and an interaction term of PGS and twin status in the regression model. Six categories were constructed, representing the following BW ranges: <2000, 2000–2500, 2501–3000, 3001–3500, 3501–4000, >4000 g. Complete regression equations can be found in the Supplementary Methods. The phenotypic variance explained, captured by *R*^2^, was used to evaluate the predictive performance of each PGS. Our main interest was to determine how well PGS derived from a large discovery population, reflecting general population numbers of twins, could predict BW in a separate target population of twins and singletons.

## Supplementary Material

BW_Supp_Fig_1_ddab121

Supplemental_Tables_Beck_ddab121

Supplementary_Methods_Beck_ddab121

## Data Access

Summary statistics for the GWAMA of BW in twins can be downloaded from the GWAS catalog website: https://www.ebi.ac.uk/gwas/ .
