# Supplementary figures and images for: Genetic meta-analysis of twin birth weight shows high genetic correlation with singleton birth weight
Source: Hum Mol Genet. 2021 May 6;30(19):1894–905. doi: 10.1093/hmg/ddab121 (PMC8444448; doi:10.1093/hmg/ddab121)

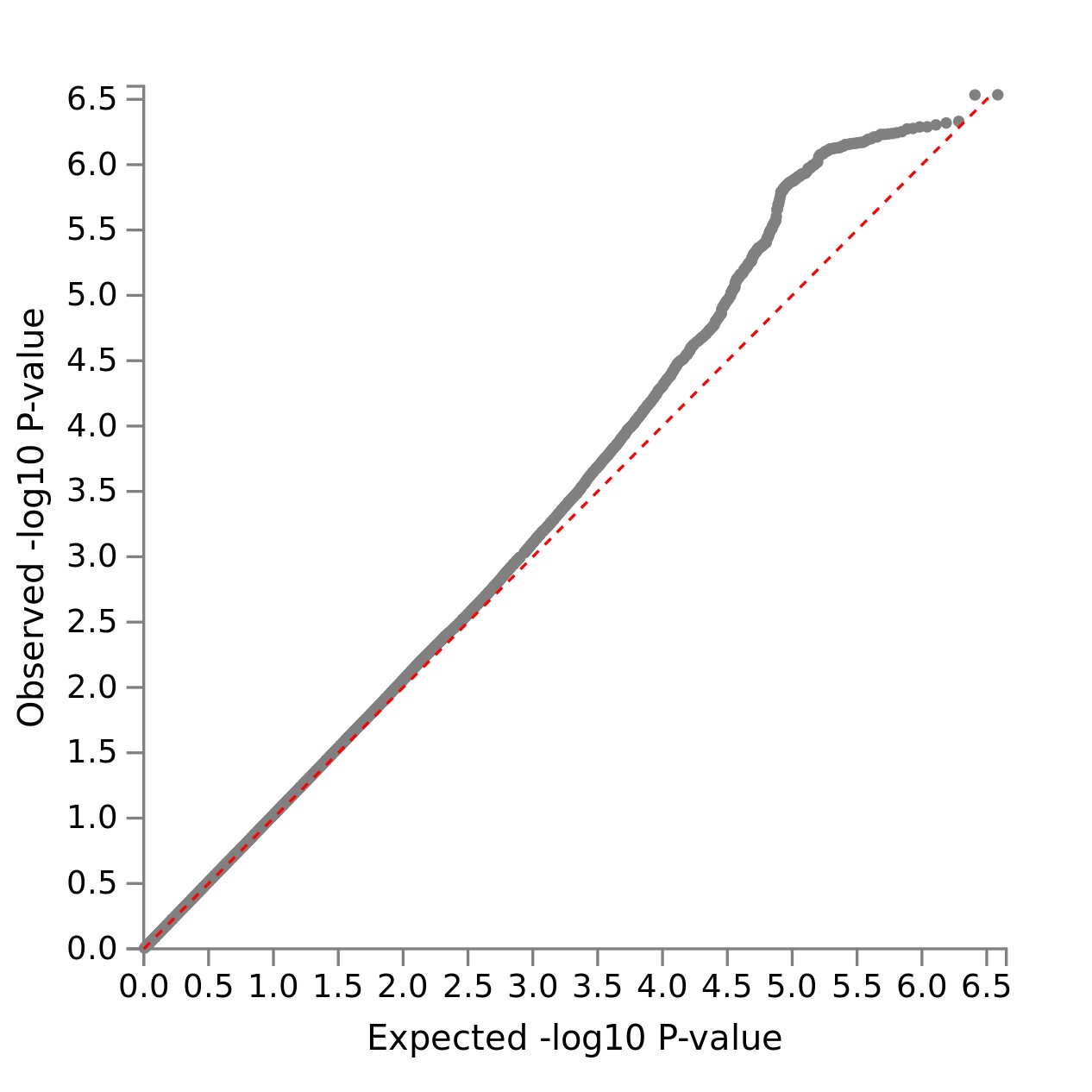

Supplement: BW_Supp_Fig_1_ddab121 [file bw_supp_fig_1_ddab121.jpeg]
