# Supplementary material for: Genetic meta-analysis of twin birth weight shows high genetic correlation with singleton birth weight
Source: Hum Mol Genet. 2021 May 6;30(19):1894–905. doi: 10.1093/hmg/ddab121 (PMC8444448; doi:10.1093/hmg/ddab121)
Supplement: Supplementary_Methods_Beck_ddab121 [file supplementary_methods_beck_ddab121.docx]

**Supplementary Methods**

*Formulas for PGS prediction*

PGS were calculated from summary statistics of a UK Biobank GWAS on BW (http://www.nealelab.is/uk-biobank/) and were used to predict BW in NTR twins and singletons.

The formula below was used to evaluate the prediction in twins and singletons separately:

**BW_raw_ ~ β_Genomic PCs_ + β_Sex_ + β_Gestational age_ + β_Year of birth_ + β_PGS_ + β_Genotyping platform_**

The formula below was used to evaluate the prediction in the entire target sample (twins and singletons) using BW category as the response. In this model, we included a main effect of twin status and an interaction term between twin status and the PGS.

For prediction in the full target sample using BW category:

**BW_category_ ~ β_Genomic PCs_ + β_Sex_ + β_Gestational age_ + β_Year of birth_ + β_PGS_ + β_Genotyping platform_ + β_Twin status_ + β_PGS_ * β_Twin status_**
